# Benefits and challenges of cervical cancer screening since the implementation of the ‘two cancer’ screening programme in China: findings from Shangyu, Zhejiang in 2019–23

**DOI:** 10.7189/jogh.15.04064

**Published:** 2025-03-07

**Authors:** Yinfang Wu, Jianqiao Luo, Danping Ye, Shujun Gao

**Affiliations:** 1 The International Peace Maternity and Child Health Hospital, School of Medicine, Shanghai Jiao Tong University, Shanghai, China; 2 Shanghai Key Laboratory of Embryo Original Diseases, Shanghai, China; 3 Zhejiang Shaoxing Shangyu Maternal and Child Health Hospital, Zhejiang, China

## Abstract

**Background:**

Cervical cancer is the fourth most common cancer in women worldwide. The World Health Organization has long targeted its elimination and stressed the need for enhanced screening coverage and improved treatment rates. The Chinese government initiated the ‘two cancer’ screening programme for cervical and breast cancer in 2009 for women aged 35–64 years, which Shangyu fully implemented in 2017. We evaluated the programme’s progress in Shangyu using data from 2019–23, aiming to suggest feasible improvements and provide general recommendations for regions facing similar challenges.

**Methods:**

We used data collected and shared by the Shaoxing Shangyu Maternal and Child Health Hospital from 2019 to 2023. The study sample included 59 201 unemployed women aged 35–64 residing in Shangyu, Shaoxing with no previous cervical cancer histories. Following international guidance, we sent their cervical samples for HPV genotyping and liquid cytology testing and asked them to receive colposcopy as needed for eventual diagnosis, which was subsequently categorised into normal, low-grade cervical intraepithelial neoplasia (CIN 1), high-grade cervical intraepithelial neoplasia (CIN 2–3), squamous cell carcinoma (SCC), adenocarcinoma in situ (AIS), and adenocarcinoma (AA). We used logistic regressions to investigate potential associations between participants’ demographic characteristics, risks of HPV positivity, and eventual diagnosis.

**Results:**

The prevalence of HPV was 11.65% between 2019 and 2023. The three most common subtypes were HPV-52, HPV-16, and HPV-59. Among those who tested positive and received colposcopy, 97.05% had a normal diagnosis, 1.68% had CIN 1, 0.64% had CIN 2–3, 5.74‱ had SCC, 0.68‱ had AIS, and 0.51‱ had AA. Participants aged 50–54 years (adjusted odds ratio (aOR) = 1.19; 95% confidence interval (CI) = 1.02–1.38), 60–64 years (aOR = 1.33; 95% CI = 1.13–1.57), and those who took birth control pills alone (aOR = 2.35; 95% CI = 1.24–4.46) were associated with an increased likelihood of being tested HPV-positive. Older ages, specifically 55–59 years (aOR = 0.53; 95% CI = 0.29–0.96) and 60–64 years (aOR = 0.46; 95% CI = 0.25–0.85), were associated with a decreased likelihood of developing CIN 2–3. Contraceptive use of intrauterine devices alone was associated with an increased likelihood of developing CIN 2–3 (aOR = 1.41; 95% CI = 1.00–1.99). Being in menopause was associated with a decreased likelihood of developing SCC (aOR = 0.2; 95% CI = 0.06–0.65).

**Conclusions:**

As the pilot city of the ‘two cancer’ screening programme, Shangyu saw a gradual yearly increase in cervical cancer screening coverage. However, lack of awareness, reluctance to receive screening and later colposcopy, and underutilisation of screening alternatives undermined further progress. Future medical services and policies should prioritise health education and target neglected groups in both rural and urban areas.

Cervical cancer, the fourth most common cancer in women, accounted for approximately 660 000 new cases and nearly 350 000 deaths worldwide in 2022 [1]. While cervical cancer affects all countries, regional differences in disease burden remain significant; for example, almost all (94%) of related deaths occur in low- and middle-income countries in 2022, while sub-Saharan Africa, Central America, and South-East Asia have the highest incidence and mortality rates [1]. With the global number of affected women projected to rise, the World Health Organization (WHO) set a goal in 2020 to almost fully eliminate cervical cancer, aiming for a threshold of four cases per 100 000 women-years by 2030 [2].

Due to its vast territorial area and massive population, China is among the regions most seriously burdened by cervical cancer [3,4]. Over the past 20 years, the morbidity and mortality rates of cervical cancer in the country have been increasing [3]. According to the Global Cancer Observatory on cervical cancer, China had 55 694 deaths and 150 659 new cases in 2022, accounting for over 20% of the global new cases [5].

It is well-established that persistent infection with oncogenic or high-risk human papillomavirus (HR-HPV) is the primary cause of precancerous and cancerous cervical dysplasia [6]. More than 90% of cervical cancers are caused by HPV, and around 70% are rooted in HPV-16, HPV-17, and HPV-18 specifically [2,7]. Therefore, cervical cancer is preventable and curable if detected early and treated properly [2], making precise screening and early evidence-based interventions crucial in reducing new cases and mortality rates [8,9]. To achieve its proposed aim and combat cervical cancer, the WHO introduced ‘90-70-90’ targets, aiming for 90% of 15-year-old girls to be vaccinated, 70% of women aged 35–45 to be screened, and 90% of those diagnosed to receive treatment by 2030 [2]. Aligning with this goal, China has made efforts to enhance cervical cancer prevention and control by launching the ‘two cancer’ screening programme for cervical and breast cancer in 2009 for women aged 35–64 in rural areas, later expanding it to urban areas in 2012 [10]. However, due to factors such as insufficient funding, the successful implementation of population-wide screening remains challenging [10,11]. Significant gaps remain in screening coverage between China and the developed countries, with the screening rates for women aged 30–49 in 2019 being at 80% in the USA, 87% in Canada, and 78% in the UK, compared to 29% in China [12]. In 2023, the screening coverage in China was only 25.7%, still lagging significantly from WHO’s 70% target [11,13]. Meanwhile, variations in screening methods and abilities, economic development levels, and cultural beliefs at the country level created disparities in cervical cancer detection rates and distributions among cities and rural/urban areas [11]. In 2018–19, cervical cancer screening rates among 20–64-year-olds were 33.4% in eastern, 28.1% in central, and 26.6% in western China; the rates in urban and rural areas were 32.2% and 26.6%, respectively [11].

Shangyu is a district of Shaoxing in the northeast of Zhejiang Province, China, with a population of 797 700 in 2023, including 151 688 women aged 35–64. Over the past ten years, it kept up with international cervical cancer prevention targets and enhanced its screening coverage. On 28 September 2012, it was selected as the national pilot area for the ‘two cancer’ screening programme implementation, under supervision from the Zhejiang Provincial Women’s Federation and Health Department. At this initial stage, women were randomly sampled from rural and urban areas. It was listed as the exclusive site for free screening in order to improve screening quality and expanding coverage in 2015, with the free screening project being fully launched in 2017. In 2021, the screening population was expanded to all women aged 35–64 in Shangyu district, with the expectation that the full district would be screened every five years.

Though multiple studies have examined the progress of ‘two cancer’ screening programmes in China and have proposed it to be beneficial for the country in the long-term, there are few analyses regarding a single city or district [10,14]. The progress of cervical cancer screening in Shangyu as the pilot site for the national programme have not yet been evaluated. Given the city’s varying development orientations and demographic compositions, we believe it is crucial to examine regions separately so as to provide evidence for the government when designing cervical cancer control strategies in China. In this study, we analysed Shangyu’s cervical cancer screening data for the 2019–23 period, aiming to evaluate the progress of the ‘two cancer’ screening programme in Shangyu and identify potential challenges the district faces. We also aimed to propose some feasible improvements for future prevention strategies and discuss the generalisability of our findings to other contexts that face similar challenges.

## METHODS

### Data and study sample

For this cross-sectional study, we used the data collected and shared by Shaoxing Shangyu Maternal and Child Health Hospital between 2019 and 2023. We included women aged 35–64 who had sexual history, were unemployed, resided in Shangyu, Shaoxing for more than 6 months, voluntarily underwent free HPV testing, had no history of total hysterectomy, and have not been diagnosed with cervical cancer. We excluded women unsuitable for gynaecological examination. Each participant was assigned a unique ID which they retained if they came in for subsequent check-ups.

### HPV genotyping and liquid cytology tests

Participants’ cervical samples were collected for the HPV genotyping test using a fluorescent polymerase chain reaction test. As confirmed by the WHO, 14 HR-HPV genotypes can be detected, namely HPV-16, 18, 31, 33, 35, 39, 45, 51, 52, 56, 58, 59, 66, and 68 [15]. If any of the above strains were detected, the participant was considered HPV positive.

For participants who tested HPV positive twice, their infections were further categorised into persistent or recurrent infection. If at least one infected HR-HPV subtype was detected in both screening tests, we considered the infection to be persistent; if the infected HR-HPV subtypes were completely different in the two screening tests, we classified it as recurrent.

For further diagnosis, we asked participants with HPV-16 and HPV-18 to receive colposcopy checks, and the others to undergo liquid cytology tests. In the latter case, cervical samples were stained using the Papanicolaou method. Based on Bethesda system criteria, we classified diagnosis results as negative intraepithelial lesion or malignancy, squamous cell abnormalities, glandular cell abnormalities, and other malignant neoplasms [16]. We further subdivided squamous cell abnormalities into atypical squamous cells of undetermined significance, low-grade squamous intraepithelial lesions, high-grade squamous intraepithelial lesions, and squamous cell carcinoma (SCC). Glandular cell abnormalities were further categorised into atypical glandular cells not otherwise specified, adenocarcinoma in situ (AIS), and adenocarcinoma (AA). Participants diagnosed with squamous cell, glandular cell abnormalities, or other malignant neoplasms were asked to receive colposcopy checks.

### Eventual diagnosis

We provided any eventual diagnoses to participants who received the colposcopy check. They were classified as normal, low-grade cervical intraepithelial neoplasia (CIN 1), high-grade cervical intraepithelial neoplasia (CIN 2–3), SCC, AIS, and AA.

Follow-up visits were recommended for patients diagnosed with CIN 1. Those with CIN 2–3 and higher dysplasia stages were asked through phone calls to make a hospital revisit appointment for further checks and treatments including cervix conisation and loop electrosurgical excision procedure. If the pathology suggests advanced cancer dysplasia, a treatment plan was formulated based on the current malignancy stage. Otherwise, patients were followed up until tested HPV-negative.

## RESULTS

### The overall prevalence of HPV infection each year

**Figure 1:**
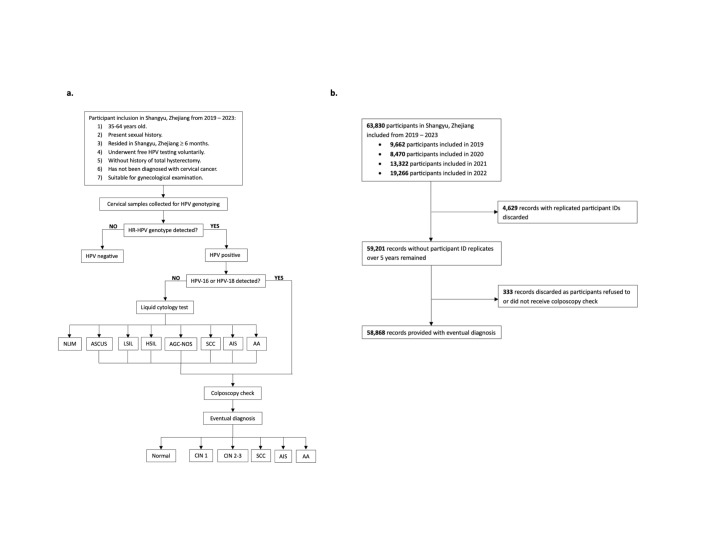
Diagnostic flowchart and sample selection process. **Panel A.** Flowchart of the inclusion criteria, screening, and diagnostics. **Panel B.** Sample selection process. AA – adenocarcinoma, AGC-NOS – atypical glandular cells not otherwise specified, AIS – adenocarcinoma in situ, ASCUS – atypical squamous cells of undetermined significance, CIN 1 – low-grade cervical intraepithelial neoplasia, CIN 2–3 – high-grade cervical intraepithelial neoplasia, HPV – human papillomavirus, HR-HPV – high-risk human papillomavirus, HSIL – high-grade squamous intraepithelial lesions, LSIL – low-grade squamous intraepithelial lesions, NLIM – negative intraepithelial lesion or malignancy, SCC – squamous cell carcinoma.

We included 63 830 records satisfying these inclusion criteria. According to existing guidance, each woman of reproductive age should have an HPV check every three years [2]. Of the 4629 patients with two testing records, we kept the latest record for those who tested negative or positive twice and the positive record was retained for those who once tested positive and once negative. This left us with 59 201 records. To further analyse the dysplasia status of HPV-positive participants, we excluded individuals who tested positive, but refused colposcopy checks. The final sample thus included 58 868 women who were tested for HPV and received colposcopy as needed from 2019 to 2023. (**Figure 1**, Panels A and B)

**Figure 2:**
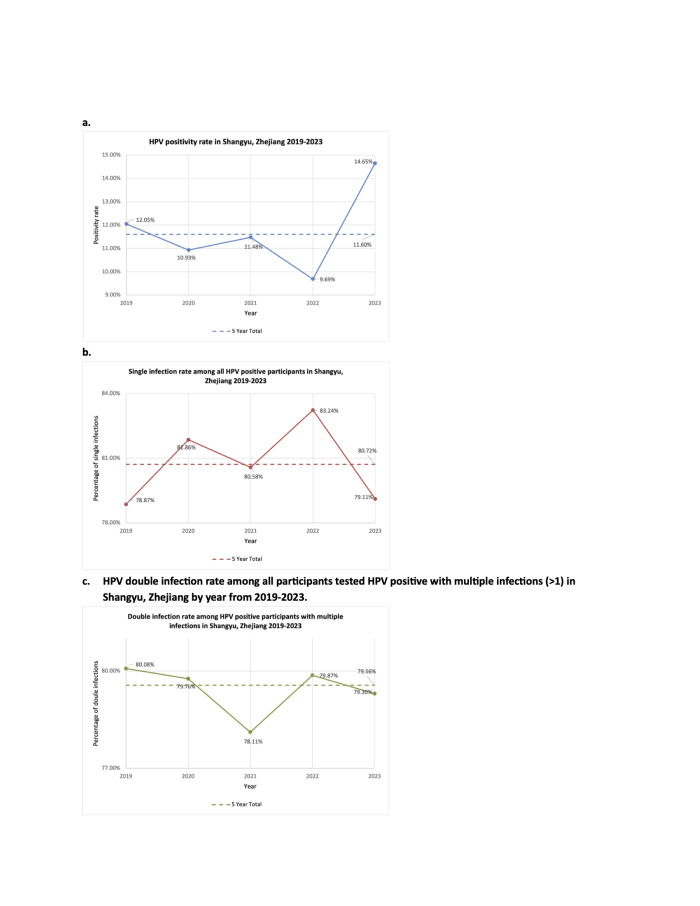
HPV positive rate, single infection rate, and double infection rate in Shangyu, Zhejiang (2019–23). **Panel A.** HPV-positive rates. Number of participants in each year: 2019 (n = 9662), 2020 (n = 8470), 2021 (n = 13 322), 2022 (n = 19 266), 2023 (n = 13 110), and five-year total (n = 59 201). **Panel B.** Single infection rates among all HPV-positive participants. Number of HPV-positive participants in each year: 2019 (n = 1164), 2020 (n = 926), 2021 (n = 1529), 2022 (n = 1867), 2023 (n = 1920), and five-year total (n = 6902). **Panel C.** Double infection rates among all HPV-positive participants with multiple infections. Number of HPV-positive participants with multiple infections in each year: 2019 (n = 246), 2020 (n = 168), 2021 (n = 297), 2022 (n = 313), 2023 (n = 401), and five-year total (n = 1331). HPV – human papillomavirus.

**Figure 3:**
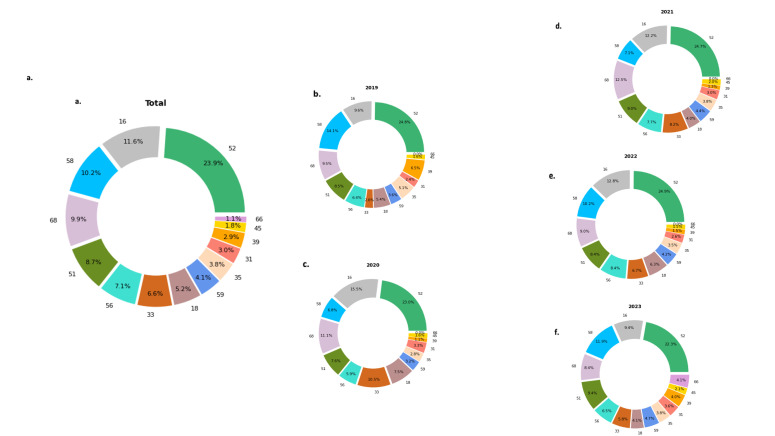
Distribution of infected HR-HPV subtypes among participants who tested HPV positive in each year. **Panel A.** Total. **Panel B.** 2019. **Panel C.** 2020. **Panel D.** 2021. **Panel E.** 2022. **Panel F.** 2023. HPV – human papillomavirus, HR-HPV – high-risk human papillomavirus.

Of the 59 201 participants in Shangyu, 0.28% were vaccinated against HPV (Table S1 in the **Online Supplementary Document**). The overall prevalence of HPV was 11.6% over the five years (**Figure 2**, Panel A). The five leading HR-HPV genotypes were HPV-52 (23.91%), HPV-16 (11.62%), HPV-58 (10.22%), HPV-68 (9.92%), and HPV-51 (8.71%) (**Figure 3**, Panel A). Among the 4629 participants with two records, 5.9% tested HPV-negative on the second check and 5% tested positive on both checks. Of the latter group, 33.8% had a recurrent infection and 67.2% had a persistent infection (Table S2 in the **Online Supplementary Document**). Of those with persistent infection, 39.2% were infected with HPV-52, 14.38% with HPV-16, and 13.73% with HPV-58 (Table S3 in the **Online Supplementary Document**).

Among those HPV-positive, 80.72% were infected with a single, 15.34% with two, and 3.94% with more than two HR-HPV subtypes (**Figure 2**, Panel B). Of those who had multiple HR-HPV infections, 42.98% were infected with HPV-52 (Table S4 in the **Online Supplementary Document**). Over 20% of multiple infections included at least one of HPV-58, HPV-16, HPV-68, and HPV-51. Among double infections, HPV-16 and HPV-52 ranked the highest (10.29%), followed by HPV-52 and HPV-58 (10.10%), HPV-51 and HPV-52 (9.07%), HPV-52 and HPV-68 (8.69%), HPV-52 and HPV-56 (8.22%) (Table S5 in the **Online Supplementary Document**).

Over the five years, the HPV prevalence was lowest in 2022 (9.69%) and highest in 2023 (14.65%) (**Figure 2**, Panel A). The most common HPV subtype in every year was HPV-52, with the prevalence exceeding 20%. Other leading HR-HPV genotypes were slightly different each year among HPV-16, HPV-33, HPV-51, HPV-52, HPV-58, and HPV-68. These HR-HPV genotypes accounted for over 65% of all infections (**Figure 3**, Panels A–F).

The percentage of single infections ranged from 78.87% in 2019 to 83.24% in 2022, that of double infections ranged from 16.67% in 2022 to 21.13% in 2019, and that of multiple infections with more than two HR-HPV subtypes ranged from 3.37% in 2022 to 4.32% in 2023 (**Figure 2**, Panels B and C). The most frequent co-infected HR-HPV subtype in multiple infections every year was HPV-52, with a prevalence of 50% in 2020 and around 40% in other years (Table S3 in the **Online Supplementary Document**).

### HPV positivity in different populations

Based on the entire five-year data, HPV-positive rates roughly increased with age, with 9.39% of participants aged 35–39 and 13.39% of those aged 60–64 testing HPV-positive (Table S1 in the **Online Supplementary Document**). With the education level increasing, HPV-positive rates tend to decrease, with 12.7% of those with primary school or lower being infected compared to 7.36% of those with college or higher. We also found lower rates among women who used condoms (8.98%) and have been through menopause (10.26%). Even though we have limited data, HPV-positive rates among those who have been vaccinated against HPV (0.09%) were significantly lower than among the unvaccinated (11.67%).

Participants’ older ages and usage of combined oral contraceptives (COCs) alone for contraception were associated with an increased likelihood of being tested HPV positive. Compared to those aged 35–39, the odds of being tested HPV positive were about 0.2 times higher for those aged 50–54 (adjusted odds ratio (aOR) = 1.19; 95% confidence interval (CI) = 1.02–1.38, *P* = 0.029) and 0.3 times higher for those aged 60–64 (aOR = 1.33; 95% CI = 1.13–1.57, *P* = 0.001) (**Table 1**). Compared to participants without contraception, the odds of being tested HPV positive were more than double for participants who took COCs alone (aOR = 2.35; 95% CI = 1.24–4.46, *P* = 0.009).

**Table 1:** Associations between participants’ demographic characteristics and risks of HPV positivity*

	HPV positivity	
	**aOR (95% CI)**	***P*-value**
**Age in years**		
35–39	ref	
40–44	0.99 (0.85–1.16)	0.905
45–49	1.13 (0.98–1.30)	0.098
50–54	1.19 (1.02–1.38)	0.029
55–59	1.14 (0.97–1.35)	0.109
60–64	1.33 (1.13–1.57)	0.001
**Education**		
Primary school or below	ref	
Middle school	0.94 (0.85–1.05)	0.264
High school	084 (0.67–1.07)	0.159
College or above	0.70 (0.43–1.13)	0.146
Do not know	1.00 (035–2.84)	0.995
**Menopause**		
No	ref	
Yes	1.09 (0.99–1.20)	0.094
**Contraception**		
No action	ref	
Condom	0.91 (0.77–1.07)	0.260
COCs	2.35 (1.24–4.46)	0.009
IUD	1.06 (0.96–1.16)	0.265
Others	1.02 (0.93–1.13)	0.617
**Pregnancy times**		
0	ref	
1	0.72 (0.45–1.17)	0.183
2	0.78 (0.48–1.25)	0.303
≥3	0.83 (0.51–1.33)	0.433
**Birth times**		
0	ref	
1	1.30 (0.80–2.11)	0.284
2	1.28 (0.79–2.08)	0.312
≥3	1.45 (0.87–2.42)	0.152

aOR – adjusted odd ratios, CI – confidence interval, COC – combined oral contraceptive (birth control pills), HPV – human papillomavirus, IUD – intrauterine device, ref – reference

*Included 59 201 participants.

### Dysplasia status

Of the 59 201 screened participants, 97.05% were with normal diagnosis, 1.68% were diagnosed with CIN 1, 0.64% with CIN 2–3, 5.74‱ with SCC, 0.68‱ with AIS, and 0.51‱ with AA (Table S6 in the **Online Supplementary Document**). Narrowing down to the 6569 HPV-positive participants with colposcopy check, 78.51% received a normal diagnosis, 15.16% were diagnosed with CIN 1, 5.71% with CIN 2–3, 51.76‱ with SCC, 6.09‱ with AIS, and 4.57‱ with AA.

Considering different HPV subtypes, HPV-16 and HPV-18 had the most dysplasia development. The leading five genotypes with higher percentages developing into CIN 1 were HPV-18, HPV-16, HPV-31, HPV-35, and HPV-39, those developing into  CIN 2–3 were HPV-16, HPV-31, HPV-33, HPV-18, and HPV-52, and those developing into SCC were HPV-16, HPV-31, HPV-18, HPV-33, and HPV-51 (Table S7 in the **Online Supplementary Document**). Of those who tested positive for HPV-16, 33.3% were diagnosed with CIN 1, 20.02% with CIN 2–3, 2.03% with SCC, 0.11% with AIS, and 0.21% with AA. Of those who tested positive for HPV-18, 35.98% were diagnosed with CIN 1, 6.54% with CIN 2–3, 0.93% with SCC, 0.3% with AIS, and 0.23% with AA. Following these two genotypes, 19.12% of those who tested positive for HPV-31 developed into CIN 1, 10.76% into CIN 2–3, and 1.59% into SCC. As the project is government-initiated, we assumed patients’ compliance upon subsequent checkups. Patients who declined appointments for further treatments were assured that they would receive further checkups in higher-level hospitals.

Combining all patients diagnosed with CIN 2–3, SCC, AIS, and AA (n = 417), the percentage of participants eventually diagnosed increased with age, with 15.8% diagnosed among those aged 35–44 years, 38.9% among those aged 45–54 years, and 45.3% among those aged 55–64 years (Table S1 in the **Online Supplementary Document**). Compared to those aged 35–39, the odds of being diagnosed with CIN 2–3 were about 0.5 times higher for those aged 55–59 (aOR = 0.53; 95% CI = 0.29–0.96, *P* = 0.036) and those aged 60–64 (aOR = 0.46; 95% CI = 0.25–0.85, *P* = 0.013) (**Table 2**). Compared to participants not using contraception, the odds of being diagnosed with CIN 2–3 were about 0.4 times higher for participants who used the intrauterine device alone (aOR = 1.41; 95% CI = 1.00–1.99, *P* = 0.049). Lastly, compared to those who have not been through menopause, the odds of being diagnosed with SCC were about 0.8 times lower for women who had been through menopause (aOR = 0.2; 95% CI = 0.06–0.65, *P* = 0.007).

**Table 2:** Associations between participants’ demographic characteristics and eventual diagnosis*

	CIN 1		CIN 2–3		SCC		AIS		AA	
	**aOR (95% CI)**	***P*-value**	**aOR (95% CI)**	***P*-value**	**aOR (95% CI)**	***P*-value**	**aOR (95% CI)**	***P*-value**	**aOR (95% CI)**	***P*-value**
**Age**										
35–39	ref		ref		ref		ref		ref	
40–44	0.998 (0.658–1.516)	0.994	0.706 (0.408–1.222)	0.213	NA		0.591 (0.036–9.614)	0.712	NA	
45–49	1.079 (0.737–1.58)	0.695	0.677 (0.41–1.117)	0.127	0.817 (0.142–4.713)	0.822	0.328 (0.02–5.331)	0.433	NA	
50–54	0.7 (0.464–1.057)	0.090	0.606 (0.353–1.037)	0.068	1.68 (0.311–9.081)	0.547	0.41 (0.025–6.771)	0.533	NA	
55–59	0.857 (0.553–1.329)	0.490	0.528 (0.29–0.959)	0.036	4.674 (0.694–31.48)	0.113	NA		0.047 (0–14411)	0.635
60–64	0.767 (0.493–1.195)	0.241	0.464 (0.253–0.852)	0.013	2.698 (0.358–20.32)	0.335	NA			
**Manopause**										
No	ref		ref		ref		ref		ref	
Yes	0.896 (0.687–1.168)	0.416	0.896 (0.606–1.324)	0.581	0.2 (0.062–0.647)	0.007	NA		NA	
**Contraception**										
No action	ref		ref		ref		ref		ref-	
Condom	0.69 (0.434–1.095)	0.115	0.769 (0.393–1.504)	0.442	1.098 (0.123–9.807)	0.933	NA		NA	
COCs	2.448 (0.578–10.36)	0.224	4.688 (0.888–24.77)	0.069						
IUD	0.779 (0.597–1.016)	0.065	1.413 (1.002–1.993)	0.049	1.101 (0.337–3.598)	0.873	0.896 (0.08–10.05)	0.929	1.932 (0.128–29.14)	0.634
Others	0.797 (0.618–1.029)	0.081	0.993 (0.675–1.46)	0.972	0.286 (0.039–2.112)	0.220	6.936 (1.638–78.79)	0.118		

AA – adenocarcinoma, AIS – adenocarcinoma in situ, aOR – adjusted odd ratios, CIN 1 – low-grade cervical intraepithelial neoplasia, CIN 2–3 – high-grade cervical intraepithelial neoplasia, COCs – combined oral contraceptives (birth control pills), HPV – human papillomavirus, IUD – intrauterine device, NA – not applicable, SCC – squamous cell carcinoma

*aORs and 95% CIs are nor presented in most AIS and AA cases analysis due to limited diagnostic cases leading to small sample sizes.

## DISCUSSION

### HPV infection pattern and low vaccination uptake

Consistent with existing studies in China and worldwide [17], we found that HPV-52 was more prevalent, but exhibited lower carcinogenic risks of developing CIN 2–3 and cervical cancer compared to HPV-16, HPV-18, and HPV-31. These carcinogenic risk differences guide subsequent clinical treatment plans differently. Individuals infected with HPV-16 and HPV-18 instantly require colposcopy checks regardless of their cytology test results, while those with HPV-52 with a negative intraepithelial lesion or malignancy cytology test results should avoid over-treatment of colposcopy and require only regular follow-up visits [18].

Our results suggested that participants’ older age (*i.e.* 50–54 and 60–64 years) was associated with an increased likelihood of HPV positivity, but decreased likelihood of CIN 2–3 in the 55–59 age group. The two age peaks for HPV infection in China are around 20–25 and 50–60 years [19]. Generally, it takes 15–20 years for an HPV infection to progress into advanced dysplasia and eventually cervical cancer, so individuals around 50–60 who tested positive are not yet likely to develop CIN 2–3 [20]. Meanwhile, women in their 50s typically have fewer sexual activities and a more stable socioeconomic status compared to younger age groups, resulting in lower risks for CIN diagnosis [21].

Contraception methods are another risk factor for cervical cancer [21], with the use of COCs being linked to a higher likelihood of HPV-positivity, as observed previously [22]. Behaviourally, COCs do not prevent skin-to-skin contact like condoms, allowing HPV transmission, while biologically, COCs disrupt women’s hormone levels, potentially triggering future HPV-related neoplasia.

Besides screening, we noted a low vaccination rate of 0.28% in Shangyu. Some possible reasons behind this include limited access, high costs, and lack of awareness. The HPV vaccine first entered the Chinese mainland market in July 2016 [23]. Due to limited supply and lack of publicity, the promotion of vaccination was ineffective in the first few years. In 2019, the coverage among women was only 3% in China and 1.81% in Zhejiang [24,25]. Additionally, the HPV vaccine is not included in the Chinese national immunisation programme, requiring individuals to pay out of pocket. This high cost and insufficient public awareness have posed challenges to HPV vaccination in China [26]. Therefore, optimising the introduction of the HPV vaccine into the national immunisation programme is crucial to address the low vaccination rate. Furthermore, the age range for 3-valent vaccine was only extended to 9–45 years in December 2020 [25], whereas the previous thresholds were 9–25 years for 2-valent, 20–40 for 4-valent, and 16–26 for 9-valent vaccines, leaving older women excluded [25]. In our study, only 13% of participants aged 35–44 were within the eligible range.

### Sustainable screening progress and challenges

Over the past five years, the screening coverage of women aged 35–64 in Shangyu reached 42.1%. Notably, this programme targeted women without a stable job, as those with stable jobs are required to complete physical examinations, including HPV screening, every year by their employers. Therefore, we speculated that the actual coverage could be much higher if the programme involved women with stable jobs, suggesting that it has been making positive progress.

The covered population gradually increased from 2019 to 2023, likely due to improved patient participation and compliance, along with the programme’s longer implementation. The limited reach in 2019 and 2020 may be attributed to the COVID-19 pandemic, since recurring lockdown policies restricted the mobility of doctors and patients. The responsibilities of medical services and professional personnel were reassigned to combat the pandemic; screening of all types of cancer was suspended worldwide, delaying coverage of the target population and early detection [26,27]. After the pandemic, screening was quickly integrated with other medical services in China, yet patients’ fear of visiting public places, particularly health facilities, still slowed recovery. This highlighted the inefficiency of the current coverage method, which relied solely on doctors reaching out to residents, resulting in a limited and unstable reach.

The number of patients screened depends on several factors, including policy changes, job shifts, and personnel arrangements. If we solely base the coverage rate on how many residents doctors can reach, the enormous inputs of labour, time, and funding are insufficient and unsustainable in the long term. Based on this, we proposed three targets for improvement: inadequate willingness from the population to actively seek screening, reluctance to undergo colposcopy when needed, and the use of a single screening method, which increases waiting times and reduces screening availability.

### Stereotypes and low awareness underlie screening reluctance

Previous research has suggested several potential barriers to cervical cancer screening. Due to the sexually transmitted nature of HPV, positive test results were associated with social and psychological burdens, with studies in developed countries showing that women may feel stigmatised, anxious, and worried that the results would affect their current relationship [28,29]. It usually takes 10–20 years for HPV infections to develop into cancer, and thus current sexual relationships cannot fully reflect women’s infection conditions [30]. At the same time, though scientists have long emphasised that sexual behaviour only increases HPV exposure and not necessarily the infection risks, misconceptions about the link persist [29]. Those misunderstandings could be the reason for women’s reluctance to go in for screening or further colposcopy checks.

Since Shangyu lies near many more economically developed cities, including Shanghai, Ningbo, and Hangzhou, most of its young generations migrated for purposes of education or more promising employment opportunities. In 2023, 31.5% of Shangyu’s population was aged >60. Conversely, those who remained in rural Shangyu held lower education levels and were more conservative. Thus, their clinging to the above stigma and low awareness of the disease further exacerbate reluctance.

In a previous study, many women stated they had more important things to worry about than attending screening if they felt healthy [29]. Like other cancers, cervical cancer shows no signs or symptoms at early stages [31]. Some patients seek screening only when experiencing vaginal bleeding or pain, by which point the dysplasia has often already advanced.

### Inefficiency of unitary screening method rendered by COVID-19

As countries faced disruptions similar to those seen in China during the COVID-19 pandemic and a slow recovery afterwards, many recognised that traditional HPV screening was insufficient, leading to the introduction of HPV self-sampling [32]. Its validity, safety, convenience, and effectiveness as a collection alternative have widely been supported in both low- and high-resource settings [33]. However, the implementation of self-sampling worldwide is challenging. Among countries with established screening programmes, only 12% recommended HPV self-sampling and only 5.6% successfully reached underscreened populations [32]. In China, however, the promotion of HPV self-sampling is still in its early stages, with few people aware of it and even fewer accepting or using it; in fact, the percentages of respondents using self-sampling are much lower than the 68% in rural El Salvador, 90% in Malaysia, and other American or European countries [32,34,35]. Most respondents unwilling to use self-sampling questioned the test accuracy, validity of specimens, and whether they followed instructions correctly [36].

Despite the low self-sampling rate in China, a study in Chengdu showed that nearly 50% of participants indicated acceptance after understanding its benefits and validity [36]. As it has a similarly low level of self-sampling, we assumed similar potential in Shangyu. As Shangyu’s primary health care institutions are only capable of sampling, we propose that they, alongside the local communities, organise routine lectures on HPV and self-sampling organised that may enable efficient sample collection and higher HPV testing rates.

### Health education in covering socioeconomic gaps

Promoting health education is crucial in the context of these concerns. Studies have emphasised that education-based interventions significantly mediate people’s behaviour and increase screening rates [36,38]. It is essential to address stereotypes linking HPV to sexual misconduct and to clarify the nature and progression of cervical cancer. Advertising posters on the street and in public transportation, promoting related videos on social platforms such as WeChat, and organising folk lectures by local communities are just some activities that can help with accelerating cervical cancer education and incorporating extra screening choices in the future.

Individuals of higher education level and socioeconomic status were previously found to have lower risks of developing cervical, breast, and colorectal cancer [39,40]. Existing research posited that education determines individuals’ access to various resources such as income, healthier lifestyles, and advanced medical services, which can help with improving their health [41. Other studies showed that people with higher education levels demonstrated higher screening rates and chances for early detection [42].

However, our logistic regression showed no significant association between participants’ education level and their likelihood of being tested HPV positive or dysplasia status. This finding could be due to several reasons. First, previous studies usually analysed data collected from patients who were voluntarily checked for HPV [3,4,19,25] In this study, data were collected by doctors reaching out to check on women residents in Shangyu. Second, most participants in our study have never undergone HPV screening before. Therefore, the factors that typically hinder patients’ access to cancer care, such as insufficient access to health services, unhealthy lifestyles, failed adherence to screening, and lower chances of early detection, might have been less significant. Aligning with a 2018–19 study [11], the prioritisation of rural screening has helped reduce the traditional disadvantages faced by rural areas. With this accomplishment, we believe that the prioritisation and initiatives of the screening programme have helped offset the disadvantages typically associated with rural areas. Few primary health care institutions are available in rural Shangyu, and most can only perform sampling, with samples sent to urban higher-level hospitals for processing. This reduces residents’ trust in and utilisation of rural facilities. Additionally, as most educated residents left rural Shangyu, those who remain tend to have less awareness of HPV and are less likely to seek screening. Only 19% of rural women now in China are screened, making the prioritisation of screening coverage for rural women is essential [41].

Conditions in urban regions have not been optimal either due to rapid urbanisation, with an increasing number of rural workers migrating to cities in search for employment. From 2013–23, the number of rural migrant workers grew from 269 million to 297.53 million [41,43]. Unfortunately, fewer than 30% of these workers have access to basic pensions or medical insurance, and around 50% lack access to health care professionals [41,43]. Collaborating with hospitals and employers to introduce special screening programmes for urban migrant workers or incorporating HPV screening into entry health checks for employees could encourage more people to get screened. Other populations, such as the unemployed, illiterate, and disabled, may face similar challenges as rural migrant workers but often receive less attention in urban settings. Identifying these groups through local residency data and linked health records could lead to improvements in cervical cancer screening and detection rates in urban regions.

## CONCLUSIONS

The ‘two cancer’ screening programme has made significant progress, but still faces challenges due to factors such as women’s lack of awareness, reluctance to attend check-ups, and the underutilisation of screening alternatives. These issues are also present in other regions of China and globally. Future research should focus on the impact of demographic and qualitative factors, such as education, socioeconomic status, and discrimination towards HPV, in shaping screening behaviours. It is crucial to educate the affected populations on the nature of the disease, the importance of screening, and the validity of screening alternatives. Additionally, targeting underserved rural women and identifying neglected minority groups in urban areas are urgent steps towards achieving the WHO’s screening goals in rapidly developing cities like Shangyu.

## Additional material


Online Supplementary Document
