# Supplementary material for: Benefits and challenges of cervical cancer screening since the implementation of the ‘two cancer’ screening programme in China: findings from Shangyu, Zhejiang in 2019–23
Source: J Glob Health. 2025 Mar 7;15:04064. doi: 10.7189/jogh.15.04064 (PMC11884646; doi:10.7189/jogh.15.04064)
Supplement: Online Supplementary Document [file jogh-15-04064-s001.pdf]

Supplement to: Wu Y, Luo J, Ye D, Gao S. Benefits and challenges of cervical cancer screening since the implementation of the 'two cancer' screening programme in China: findings from Shangyu, Zhejiang in 2019-23. J Glob Health. 2025;15:04064.

ONLINE SUPPLEMENTARY DOCUMENT

Benefits and Challenges of Cervical Cancer Screening since Implementation of "two cancer" Screening Program in China: Finding from Shangyu, Zhejiang in 2019 – 2023.

Yinfang Wu B.S.[1,2,3];

Jiaoqiao Luo B.S.[3],

Danping Ye B.S.[3],

Shujun Gao, Ph.D.[1,2]†

† Correspondence:

Shujun Gao, Shanghai Jiao Tong University, The International Peace Maternity and Child Health Hospital, School of Medicine, Shanghai Jiao Tong University, Shanghai 200030, China; Shanghai Key Laboratory of Embryo Original Diseases, Shanghai 200030, China; shujun_gao@shsmu.edu.cn.

1 The International Peace Maternity and Child Health Hospital, School of Medicine, Shanghai Jiao Tong University, Shanghai 200030, China

2 Shanghai Key Laboratory of Embryo Original Diseases, Shanghai 200030, China

3 Zhejiang Shaoxing Shangyu Maternal and Child Health Hospital, Zhejiang, China

Supplementary Material 1. Characteristics of entire participants, participants who tested HPV positive, and participants who received colposcopy.

Covariants	No. (%)								
	N (n = 59201)	Women tested HPV positive (n = 6902)	Eventual Diagnosis						
			Normal[a] (n = 57454)	CIN 1[b] (n = 997)	CIN 2-3[b] (n = 376)	SCC[b] (n = 34)	AIS[b] (n = 4)	AA[b] (n = 3)	CIN 2+/SCC/AIS/AA (n = 417)
Age									
35-39	3111 (5.25)	292 (4.23)	3014 (5.25)	45 (4.51)	28 (7.45)	2 (5.88)	1 (25)	2 (66.67)	33 (7.91)
40-44	4615 (7.8)	439 (6.36)	4481 (7.8)	71 (7.12)	32 (8.51)	0 (0)	1 (25)	0 (0)	33 (7.91)
45-49	7701 (13.01)	833 (12.07)	7448 (12.96)	147 (14.74)	56 (14.89)	4 (11.76)	1 (25)	0 (0)	61 (14.63)
50-54	13483 (22.77)	1578 (22.86)	13115 (22.83)	198 (19.86)	92 (24.47)	8 (23.53)	1 (25)	0 (0)	101 (24.22)
55-59	16290 (27.52)	1885 (27.31)	15825 (27.54)	281 (28.18)	89 (23.67)	13 (38.24)	0 (0)	1 (33.33)	103 (24.7)
60-64	14001 (23.65)	1875 (27.17)	13571 (23.62)	255 (25.58)	79 (21.01)	7 (20.59)	0 (0)	0 (0)	86 (20.62)
Education									
Primary school or below	943 (1.59)	95 (1.38)	3530 (6.14)	60 (6.02)	27 (7.18)	1 (2.94)	1 (25)	0 (0)	29 (6.95)
Middle School	258 (0.44)	19 (0.28)	52715 (91.75)	923 (92.58)	340 (90.43)	33 (97.06)	3 (75)	3 (100)	379 (90.89)
High School	35 (0.06)	4 (0.06)	920 (1.6)	281 (28.18)	9 (2.39)	0 (0)	0 (0)	0 (0)	9 (2.16)
College or above	3650 (6.17)	466 (6.75)	254 (0.44)	3 (0.3)	0 (0)	0 (0)	0 (0)	0 (0)	0 (0)
Don't know	54315 (91.75)	6318 (91.54)	35 (0.06)	0 (0)	0 (0)	0 (0)	0 (0)	0 (0)	0 (0)
Manopause									
No	17941 (30.31)	1841 (26.67)	17395 (30.28)	291 (29.19)	135 (35.9)	12 (35.29)	4 (100)	2 (66.67)	153 (36.69)
Yes	41260 (69.69)	5061 (73.33)	40059 (69.72)	706 (70.81)	241 (64.1)	22 (64.71)	0 (0)	1 (33.33)	264 (63.31)
Contraception									
No action	45769 (77.31)	5387 (78.05)	44427 (77.33)	803 (80.54)	273 (72.61)	28 (82.35)	2 (50)	2 (66.67)	305 (73.14)
Condom	2060 (3.48)	185 (2.68)	2014 (3.51)	25 (2.51)	11 (2.93)	1 (2.94)	0 (0)	0 (0)	12 (2.88)
COCs	56 (0.09)	12 (0.17)	49 (0.09)	3 (0.3)	2 (0.53)	0 (0)	0 (0)	0 (0)	2 (0.48)
IUD	6186 (10.45)	673 (9.75)	5985 (10.42)	90 (9.03)	59 (15.69)	4 (11.76)	1 (25)	1 (33.33)	65 (15.59)
Others	5130 (8.67)	645 (9.35)	4979 (8.67)	76 (7.62)	31 (8.24)	1 (2.94)	1 (25)	0 (0)	33 (7.91)
Pregnancy times									
0	582 (0.98)	67 (0.97)	571 (0.99)	5 (0.5)	1 (0.27)	1 (2.94)	0 (0)	0 (0)	2 (0.48)

1	11148 (18.83)	1192 (17.27)	10835 (18.86)	189 (18.96)	64 (17.02)	7 (20.59)	1 (25)	1 (33.33)	73 (17.51)
2	22819 (38.54)	2644 (38.31)	22158 (38.57)	345 (34.6)	145 (38.56)	17 (50)	2 (50)	2 (66.67)	166 (39.81)
>=3	24652 (41.64)	2999 (43.45)	23890 (41.58)	458 (45.94)	166 (44.15)	9 (26.47)	1 (25)	0 (0)	176 (42.21)
Brith times									
0	604 (1.02)	64 (0.93)	589 (1.03)	8 (0.8)	1 (0.27)	0 (0)	0 (0)	0 (0)	1 (0.24)
1	35415 (59.82)	4030 (58.39)	34379 (59.84)	583 (58.48)	230 (61.17)	18 (52.94)	2 (50)	3 (100)	253 (60.67)
2	22010 (37.18)	2650 (38.39)	21357 (37.17)	381 (38.21)	132 (35.11)	16 (47.06)	2 (50)	0 (0)	150 (35.97)
>=3	1172 (1.98)	158 (2.29)	1129 (1.97)	25 (2.51)	13 (3.46)	0 (0)	0 (0)	0 (0)	13 (3.12)
Family history of tumor									
No	50708 (85.65)	5822 (84.35)	49234 (85.69)	820 (82.25)	319 (84.84)	24 (70.59)	3 (75)	3 (100)	349 (83.69)
Yes	3137 (5.3)	385 (5.58)	3042 (5.29)	55 (5.52)	14 (3.72)	1 (2.94)	0 (0)	0 (0)	15 (3.6)
Missing Answer	5356 (9.05)	695 (10.07)	5178 (9.01)	122 (12.24)	43 (11.44)	9 (26.47)	1 (25)	0 (0)	53 (12.71)
HPV Vaccination									
Not vaccinated	59033 (99.72)	6887 (99.78)	57291 (99.72)	995 (99.8)	375 (99.73)	34 (100)	4 (100)	3 (100)	416 (99.76)
Vaccinated	168 (0.28)	15 (0.22)	163 (0.28)	2 (0.2)	1 (0.27)	0 (0)	0 (0)	0 (0)	1 (0.24)

Abbreviation: COCs, combined oral contraceptives (birth control pills); IUD, intrauterine device.

a. Included participants who were tested HPV negative and participants who received colposcopy with eventual diagnosis of normal.

b. Included participants who received colposcopy with an eventual diagnosis of abnormalities (CIN 1/CIN 2-3/SCC/AIS/AA).

Supplementary Material 2. HPV test results, infection types, and changes of infected HR-HPV subtypes among participants with replicate records.

HPV test results	Participants with replicate records (n = 4629), No. (%)
(-/-)	3797 (82.03)
(-/+)	328 (7.09)
(+/-)	273 (5.9)
(+/+)	231 (4.99)
Recurrent infection	78 (33.77)
Persistent infection	153 (66.23)
No infected HR-HPV sybtype change	89 (58.17)
Changed infected HR-HPV sybtypes	64 (41.83)

Abbreviation: (-/-), tested HPV negative on both checks; (-/+) tested HPV negative on the first check and positive on the second check; (+/-) tested HPV positive on the first check and negative on the second check; (+/+), tested HPV positive on both checks.

Supplementary Material 3. Infected percentage of certain type of HR-HPV among participants who established persistent infection and multiple infections.

HR-HPV subtype	Participants with persistent infections (n = 153)	Participants with multiple infections, No. (%)					
		2019 (n = 246)	2020 (n = 168)	2021 (n = 297)	2022 (n = 313)	2023 (n = 401)	Total (n = 1331)
52	60 (39.22)	112 (45.53)	84 (50)	129 (43.43)	131 (41.85)	150 (37.41)	572 (42.98)
16	22 (14.38)	44 (17.89)	42 (25)	87 (29.29)	76 (24.28)	91 (22.69)	319 (23.97)
58	21 (13.73)	74 (30.08)	32 (19.05)	54 (18.18)	90 (28.75)	105 (26.18)	329 (24.72)
51	15 (9.8)	57 (23.17)	36 (21.43)	74 (24.92)	60 (19.17)	88 (21.95)	288 (21.64)
68	14 (9.15)	48 (19.51)	37 (22.02)	85 (28.62)	63 (20.13)	77 (19.2)	290 (21.79)
35	9 (5.88)	38 (15.45)	17 (10.12)	41 (13.8)	34 (10.86)	43 (10.72)	156 (11.72)
56	8 (5.23)	42 (17.07)	26 (15.48)	64 (21.55)	68 (21.73)	68 (16.96)	254 (19.08)
33	5 (3.27)	17 (6.91)	42 (25)	54 (18.18)	51 (16.29)	63 (15.71)	218 (16.38)
59	5 (3.27)	15 (6.1)	14 (8.33)	30 (10.1)	38 (12.14)	43 (10.72)	133 (9.99)
39	4 (2.61)	42 (17.07)	5 (2.98)	9 (3.03)	12 (3.83)	41 (10.22)	92 (6.91)
31	3 (1.96)	12 (4.88)	16 (9.52)	17 (5.72)	22 (7.03)	34 (8.48)	97 (7.29)
45	3 (1.96)	12 (4.88)	5 (2.98)	13 (4.38)	16 (5.11)	23 (5.74)	64 (4.81)
18	1 (0.65)	31 (12.6)	26 (15.48)	24 (8.08)	44 (14.06)	40 (9.98)	156 (11.72)
66	0 (0)	0 (0)	0 (0)	0 (0)	0 (0)	41 (10.22)	41 (3.08)

Abbreviation: HR-HPV, high-risk human papillomavirus.

Supplementary Material 4. Distribution of infected HR-HPV subtypes among HPV-positive participants and participants who were infected with multiple HR-HPV subtypes.

HR-HPV subtype	No. (%)	
	All HPV positive participants (n = 8580)	HPV positive participants with multiple HR-HPV subtype infections (n = 1331)
52	2045 (23.83)	572 (42.98)
16	1010 (11.77)	319 (23.97)
58	866 (10.09)	329 (24.72)
68	847 (9.87)	290 (21.79)
51	742 (8.65)	288 (21.64)
56	616 (7.18)	254 (19.08)
33	590 (6.88)	218 (16.38)
18	452 (5.27)	156 (11.72)
59	358 (4.17)	133 (9.99)
35	314 (3.66)	156 (11.72)
31	263 (3.07)	97 (7.29)
39	225 (2.62)	92 (6.91)
45	152 (1.77)	64 (4.81)
66	100 (1.17)	41 (3.08)

Abbreviation: HR-HPV, high-risk human papillomavirus.

Supplementary Material 5. Distribution of infected HR-HPV subtype combinations among HPV-positive participants who were infected with two HR-HPV subtypes.

Double Infected HR-HPV subtypes	HPV positive participants with double HR-HPV subtype infections (n = 1059), No. (%)
66+68	7 (0.66)
59+68	30 (2.83)
59+66	2 (0.19)
58+68	54 (5.1)
58+66	3 (0.28)
58+59	21 (1.98)
56+68	32 (3.02)
56+66	6 (0.57)
56+59	23 (2.17)
56+58	45 (4.25)
52+68	92 (8.69)
52+66	8 (0.76)
52+59	42 (3.97)
52+58	107 (10.1)
52+56	87 (8.22)
51+68	46 (4.34)
51+66	10 (0.94)
51+59	21 (1.98)
51+58	45 (4.25)
51+56	36 (3.4)
51+52	96 (9.07)
45+68	12 (1.13)
45+66	1 (0.09)
45+59	5 (0.47)
45+58	11 (1.04)
45+56	8 (0.76)
45+52	11 (1.04)
45+51	12 (1.13)
39+68	10 (0.94)
39+66	5 (0.47)
39+59	5 (0.47)
39+58	12 (1.13)
39+56	6 (0.57)
39+52	31 (2.93)

39+51	15 (1.42)
39+45	1 (0.09)
35+68	25 (2.36)
35+66	1 (0.09)
35+59	9 (0.85)
35+58	26 (2.46)
35+56	22 (2.08)
35+52	50 (4.72)
35+51	31 (2.93)
35+45	6 (0.57)
35+39	6 (0.57)
33+68	26 (2.46)
33+66	3 (0.28)
33+59	6 (0.57)
33+58	27 (2.55)
33+56	28 (2.64)
33+52	57 (5.38)
33+51	37 (3.49)
33+45	4 (0.38)
33+39	6 (0.57)
33+35	27 (2.55)
31+68	18 (1.7)
31+66	3 (0.28)
31+59	7 (0.66)
31+58	21 (1.98)
31+56	7 (0.66)
31+52	32 (3.02)
31+51	13 (1.23)
31+45	1 (0.09)
31+39	2 (0.19)
31+35	8 (0.76)
31+33	11 (1.04)
18+68	16 (1.51)
18+66	2 (0.19)
18+59	18 (1.7)
18+58	37 (3.49)
18+56	19 (1.79)

18+52	49 (4.63)
18+51	18 (1.7)
18+45	11 (1.04)
18+39	5 (0.47)
18+35	14 (1.32)
18+33	11 (1.04)
18+31	13 (1.23)
16+68	47 (4.44)
16+66	8 (0.76)
16+59	21 (1.98)
16+58	55 (5.19)
16+56	53 (5)
16+52	109 (10.29)
16+51	38 (3.59)
16+45	7 (0.66)
16+39	8 (0.76)
16+35	22 (2.08)
16+33	48 (4.53)
16+31	12 (1.13)
16+18	14 (1.32)

Abbreviation: HR-HPV, high-risk human papillomavirus.

Supplementary Material 6. Eventual diagnosis among all participants and HPV-positive participants who received colposcopy.

Diagnosis	No. (%)	
	All participants (n = 59201)	Participants who received colposcopy (n = 8580)
Normal	57454 (97.05)	5157 (78.51)
CIN 1	997 (1.68)	996 (15.16)
CIN 2-3	376 (0.64)	375 (5.71)
AIS	4 (0.01)	4 (0.06)
AA	3 (0.01)	3 (0.05)
SCC	34 (0.06)	34 (0.52)

Abbreviation: CIN 1, low-grade cervical intraepithelial neoplasia; CIN 2-3, high-grade cervical intraepithelial neoplasia; SCC, squamous cell carcinoma; AIS, adenocarcinoma in situ; AA, adenocarcinoma.

Supplementary Material 7. Distribution of infected HR-HPV subtypes among participants who received colposcopy checks and received eventual diagnoses.

Diagnosis	HR-HPV Subtype, No. (%)													
	**16 (n = 934)**	**35 (n = 295)**	**31 (n = 251)**	**18 (n = 428)**	**33 (n = 549)**	**45 (n = 147)**	**58 (n = 833)**	**39 (n = 216)**	**52 (n = 1954)**	**51 (n = 701)**	**56 (n = 588)**	**66 (n = 94)**	**59 (n = 346)**	**68 (n = 821)**
CIN 1	311 (33.3)	47 (15.93)	48 (19.12)	154 (35.98)	67 (12.2)	21 (14.29)	119 (14.29)	32 (14.81)	260 (13.31)	100 (14.27)	60 (10.2)	12 (12.77)	12 (3.47)	92 (11.21)
CIN 2-3	187 (20.02)	9 (3.05)	27 (10.76)	28 (6.54)	42 (7.65)	4 (2.72)	43 (5.16)	1 (0.46)	103 (5.27)	16 (2.28)	7 (1.19)	2 (2.13)	2 (0.58)	16 (1.95)
SCC	19 (2.03)	1 (0.34)	4 (1.59)	4 (0.93)	4 (0.73)	0 (0)	4 (0.48)	1 (0.46)	4 (0.2)	4 (0.57)	0 (0)	0 (0)	0 (0)	1 (0.12)
AIS	1 (0.11)	0 (0)	0 (0)	3 (0.7)	0 (0)	0 (0)	1 (0.12)	0 (0)	1 (0.05)	0 (0)	0 (0)	0 (0)	0 (0)	0 (0)
AA	2 (0.21)	0 (0)	0 (0)	1 (0.23)	0 (0)	0 (0)	0 (0)	0 (0)	0 (0)	0 (0)	0 (0)	0 (0)	0 (0)	0 (0)

Abbreviation: HR-HPV, high-risk human papillomavirus; CIN 1, low-grade cervical intraepithelial neoplasia; CIN 2-3, high-grade cervical intraepithelial neoplasia; SCC, squamous cell carcinoma; AIS, adenocarcinoma in situ; AA, adenocarcinoma.
